# Biocompatible, Biodegradable, and Antimicrobial Food Packaging Film from Polylactic Acid and Biogenic Vaterite CaCO_3_-Ag Hybrid

**DOI:** 10.3390/polym17101345

**Published:** 2025-05-15

**Authors:** Mohammad Hossein Azarian, Kitti Yuwawech, Waraporn Tanthanuch, Tiraporn Junyusen, Jatuphorn Wootthikanokkhan, Wimonlak Sutapun

**Affiliations:** 1 Research Centre for Biocomposite Materials for Medical, Agricultural and Food Industry, Suranaree University of Technology, Nakhon Ratchasima 30000, Thailand; mh_azarian@yahoo.com; 2 Materials Technology Program, School of Energy, Environment and Materials, King Mongkut’s University of Technology Thonburi, Bangkok 10140, Thailand; kiay_kitti@hotmail.com (K.Y.); jatuphorn.woo@kmutt.ac.th (J.W.); 3 Synchrotron Light Research Institute (Public Organization), 111 University Avenue, Muang District, Nakhon Ratchasima 30000, Thailand; waraporn@slri.or.th; 4 School of Agricultural Engineering, Institute of Engineering, Suranaree University of Technology, Nakhon Ratchasima 30000, Thailand; tirapo@sut.ac.th; 5 School of Polymer Engineering, Suranaree University of Technology, Nakhon Ratchasima 30000, Thailand

**Keywords:** active packaging, vaterite, CaCO_3_-Ag hybrid, polylactic acid, biocompatible, antimicrobial

## Abstract

Developing biocompatible and biodegradable materials for food packaging is crucial for addressing environmental concerns and ensuring food safety. In this study, we present a novel food packaging film composed of poly(lactic acid) (PLA) and biogenic vaterite CaCO_3_-Ag hybrid microspheres. A non-solution technique was employed to prepare these films, ensuring the sustainability and simplicity of the production process. X-ray diffraction and infrared spectroscopy analyses confirmed the stability and compatibility of the vaterite CaCO_3_-Ag microspheres within the PLA matrix. Cytotoxicity tests using human dermal fibroblast cells demonstrated complete biocompatibility of the films, even at high concentrations. Antimicrobial efficacy was assessed through minimum inhibitory concentration (MIC) testing, which demonstrated that PLA film containing 7 wt% vaterite CaCO_3_-Ag hybrids effectively inhibited both gram-positive and gram-negative bacteria at concentrations as low as ≤0.067 g/mL. Mechanical testing showed that the modulus and strength of PLA film increased significantly with the embedding of 5 wt% of vaterite CaCO_3_-Ag hybrid, reaching a maximum of 5.63 ± 1.51 GPa and 48.07 ± 13.81 MPa, respectively. Thermal analysis indicated improved thermal stability with the addition of the microspheres. Synchrotron X-ray absorption spectroscopy confirmed the stability of the vaterite structure and the presence of both Ag^0^ and Ag^+^ species after embedding in PLA matrix. The composite films exhibited improved oxygen and water vapor barrier properties, making them suitable for packaging applications. These findings highlight the potential of PLA-vaterite CaCO_3_-Ag hybrid films as sustainable and effective food packaging materials.

## 1. Introduction

Bacterial contamination and resulting food spoilage remain significant challenges, highlighting the need for effective packaging solutions. Active food packaging that interacts with the environment to extend the shelf life of food products has emerged as a promising approach. However, many conventional active packaging materials are derived from non-biodegradable and non-biocompatible sources, leading to environmental concerns. In response, there is a growing interest in developing active food packaging films from biodegradable and biocompatible composite materials. These materials not only offer the necessary functionality to improve food preservation but also align with sustainability goals, minimizing environmental impact and contributing to an eco-friendlier and sustainable packaging industry [1,2,3,4].

Biodegradable thermoplastics, including poly(lactic acid) (PLA), polyhydroxybutyrate (PHB), and poly(butylene succinate) (PBS), are increasingly used in antimicrobial active packaging due to their sustainability and food compatibility. PLA stands out as the most cost-effective option, sourced from renewable resources like corn starch or sugarcane, which are abundant and affordable [5]. Moreover, PLA production processes are well-established and optimized. Recent studies have extensively explored PLA films for food packaging. For example, Yang et al. [6] developed biodegradable films with antimicrobial properties using poly(butylene adipate-co-terephthalate) (PBAT), PLA, and sodium dehydroacetate-loaded diatomite, showing effective antibacterial effects (>90% for *E. coli* and >85% for *S. aureus*) and preservation of banana fruit quality during storage. Similarly, Ordoñez et al. [7] investigated active packaging with PLA, ferulic, and cinnamic acids, designing three-layered films (PLA/starch/PLA) with improved barrier properties using film spraying and electrospinning techniques. Both films inhibited *E. coli* and *L. innocua*, suggesting their potential as sustainable alternatives to traditional non-biodegradable packaging films.

Different types of additives have been incorporated into PLA polymer to serve as antimicrobial agents for the food packaging application, including AgNPS [8], ZnO NPs [9,10,11], thymol [12], carvacrol [13], curcumin [14], nisin [15], tea tree oil [16], triclosan [17], benzalkonium chloride [18], and polyhexamethylene biguanide (PHMB) [19]. While numerous studies have explored both synthetic and natural additives to impart antimicrobial properties to PLA, there has been no report on the use of biogenic CaCO_3_-Ag hybrid material as an antimicrobial agent incorporated into the PLA polymer matrix for food packaging applications. Furthermore, most existing studies have employed the solution casting approach, which is not favorable for mass industrial production and relies on non-polar solvents like chloroform that are carcinogenic and environmentally unfriendly. Therefore, in this study, we have approached the problem through melt blending as an alternative method for commercialized production.

Implementing biogenic waste, such as CaCO_3_, not only reduces waste and promotes environmental sustainability but also improves the mechanical and thermal properties of PLA. This integration of biogenic CaCO_3_ enhances the functionality of composite materials, offering a sustainable approach to advanced material development. Biogenic CaCO_3_, sourced from natural substances such as eggshells, has garnered significant attention due to its biocompatibility and waste management potential [20,21]. Moreover, utilizing biogenic vaterite CaCO_3_ as a carrier for AgNPs provides exceptional antimicrobial properties, making it well-suited for various applications, including antimicrobial coatings [22], biomedical uses [20], and active food packaging [23].

The combination of vaterite CaCO_3_ as a carrier and stabilizer for AgNPs, along with the mechanical stability, controlled release capability, and biocompatibility offered by PLA film, enhances the effectiveness of these materials for active food packaging. This research aims to demonstrate the potential of using a biogenic vaterite CaCO_3_-Ag hybrid as a sustainable approach for developing antimicrobial and biodegradable composite materials through melt mixing, rather than conventional solution mixing, by employing a micro-compounding technique. By micro-compounding the hybrid into PLA, the goal is to enhance the antimicrobial properties of PLA for active food packaging applications, with the potential for large-scale production. 

## 2. Experimental Details

### 2.1. Materials

Chicken eggshell wastes were obtained from a local bakery shop near Suranaree University of Technology (SUT) in Thailand, Nakhon Ratchasima. Biogenic vaterite CaCO_3_-Ag hybrid microspheres were obtained as described in our previous publication [24] with PSD and D_m_ of 3−3.6 um and 3.3 um, respectively. The commercial PLA grade (4043D) was purchased from NatureWorks (Bangkok, Thailand), and ethylene terpolymer impact modifier (Elvaloy^®^ PTW, Orlando, FL, USA) was obtained from Innovation Group (Bangkok, Thailand) Ltd. The Elvaloy^®^ PTW is used to enhance the toughness and flexibility of PLA by improving its impact resistance and ductility. The PLA and impact modifier were dried overnight at 60 °C prior to the micro-compounding process.

### 2.2. Micro-Compounding and Moulding Process

The samples (PLA granules, PLA granules mixed with impact modifier, and PLA granules mixed with impact modifier and different wt% of vaterite CaCO_3_-Ag hybrid) were fed into a hopper of a micro-compounder (Thermo Scientific HAAKE Mini CTW, Waltham, MA, USA). The barrel temperature, mixing time, and screw rotating speed used were 150 °C, 3 min, and 85 rpm, respectively. The obtained PLA-embedded vaterite CaCO_3_-Ag hybrid ribbons were ground prior to preparing the test specimens of the composites by compression molding. The compression molding was performed at 175 °C for 10 min. The obtained films were labelled as IMPLA (PLA with 5 wt% impact modifier), IMPLA2 (PLA with 5 wt% impact modifier and 2 wt% vaterite CaCO_3_-Ag hybrid microspheres), IMPLA5 (PLA with 5 wt% impact modifier and 5 wt% vaterite CaCO_3_-Ag hybrid microspheres), and IMPLA7 (PLA with 5 wt% impact modifier and 7 wt% vaterite CaCO_3_-Ag hybrid microspheres). The sample formulations and their sample codes are illustrated in Table 1.

### 2.3. Characterization

#### 2.3.1. Scanning Electron Microscopy (SEM) and Energy Dispersive X-Ray Spectroscopy (EDS)

The film morphology, including surface and cryogenic cross-section (using liquid nitrogen), was examined using a Field Emission Scanning Electron Microscope (FE-SEM) (JEOL JSM 7800F, JEOL Ltd., Tokyo, Japan) equipped with energy dispersive X-ray spectroscopy (EDX). Prior to SEM analysis, the samples were coated with a thin layer of gold. Statistical image processing included measuring the dimensions of the microspheres using DigiMizer software version 6.4.5 (Media Cybernetics, Rockville, MD, USA).

#### 2.3.2. X-Ray Diffraction (XRD)

The structural phase study of the vaterite CaCO_3_-Ag hybrid and the films was conducted by powder X-ray diffraction (XRD) analyzer in 2θ range of 5–70° using XRD (Model: Bruker D8 ADVANCE, Bruker, Billerica, MA, USA) at the voltage of 40 kV, a current of 40 mA, and Cu Kα (1.5606 Å) radiation source.

#### 2.3.3. Attenuated Total Reflection Infrared Spectroscopy (ATR-FTIR)

Attenuated total reflection (ATR) spectra of vaterite CaCO_3_-Ag hybrid and the films were recorded from a spectrophotometer (Bruker Tensor 27, Bruker) at the ambient temperature. The samples were scanned over wavenumbers ranging from 4000 to 650 cm^−1^ with a resolution of 4 cm^−1^. All the spectra were collected after an average of 16 scans for each specimen.

#### 2.3.4. Synchrotron X-Ray Absorption Spectroscopy (XAS)

Calcium K-edge X-ray Absorption Spectroscopy (XAS) measurements were conducted at Beamline 5.2 (BL5.2) of the Siam Photon Laboratory, Synchrotron Light Research Institute (Public Organization), Nakhon Ratchasima, Thailand. The beamline operates within an energy range of 1.8–13 keV. Energy scans were performed using a double crystal monochromator (DCM) equipped with Ge(220) crystals. The measurements were carried out in fluorescent mode, with the absorption edge (E0) set at 4039 eV of Ca-K edge XANES. For energy calibration, CaCO_3_ was utilized to align the white line region accurately. The IFEFFIT software version 0.9.26, which comprises Athena and Artemis, was employed for spectral normalization and EXAFS fitting.

#### 2.3.5. Thermogravimetric Analysis (TGA) and Differential Scanning Calorimetry (DSC)

The thermal analysis was conducted using TGA (NETZSCH, Selb, Germany, TG 209 F3 Tarsus^®^). The samples were heated from 30 to 900 °C at a heating rate of 10 °C/min under nitrogen flow with a rate of 20 mL/min. The DSC was conducted using METTLER TOLEDO (STARe DSC 3 system), Columbus, Ohio, USA from 0 to 200 °C at a heating rate of 10 °C/min under nitrogen flow. The degree of crystallinity (X_c_) was calculated from the following formula [25]:

(1) $$X_{c}=\frac{{∆H}_{m}}{{∆H}_{m}^{0}}\frac{1}{Xp}$$

where ∆H_m_ is the heat of melting of the PLA polymer, ${∆H}_{m}^{0}$ is the heat required for melting of 100% crystalline PLA polymer (93.60 J/g) [26], and Xp is the PLA polymer weight fraction.

#### 2.3.6. Universal Test

The specimens for tensile measurement were prepared by molding the films with 0.4 mm thickness into a rectangular shape according to ASTM D882-02 by compression molding [27]. The measurement was performed using the universal testing machine (Instron 5569, Norwood, MA, USA) under a 5 kN load cell. The measurement was repeated more than three times for each sample. The toughness was calculated from the area under the stress-strain curve.

#### 2.3.7. Nanoindentation

Nanomechanical measurements were conducted using a NanoTest indenter (Micro Materials, Wrexham, UK) on platform 3, employing a Berkovich triangular diamond pyramid indenter in accordance with ASTM E2546-07 standards [28] A 60 mN loading rate was applied for 10 s before unloading. Ten indents were made on each sample at randomly selected locations to determine average hardness and elastic modulus values.

#### 2.3.8. Cell Cytotoxicity Assay

The MTT assay was employed to assess the impact of PLA polymer, both before and after embedding with vaterite CaCO_3_-Ag hybrid, on the viability of Human Dermal Fibroblast (HDF) cells. HDF cells were seeded at a density of 7 × 10^4^ cells per well in 96-well plates. The samples underwent UV sterilization for 15 min. Subsequently, the samples were immersed in a culture medium (DMEM with 4.5 g/L glucose, SA) containing 1% penicillin/streptomycin and incubated overnight at 25 °C. After incubation, the cells were exposed to the prepared samples at a concentration of 50 mg/mL for 24 h. Following this, MTT solution was added to each well and incubated at 37 °C in the dark for 4 h. Formazan crystals formed were dissolved with DMSO, and the absorbance was measured at 570 nm using a microplate reader (BMG Labtech, Ortenberg, Germany). All experiments were conducted in triplicate, with results reported as mean ± standard deviation. Statistical significance was determined using a student’s *t*-test (SPSS version 26.0, SPSS Inc., Chicago, IL, USA), with * *p* < 0.05, ** *p* < 0.01, and *** *p* < 0.001 indicating significant differences.

#### 2.3.9. Disk-Diffusion Susceptibility Test

Antimicrobial properties were evaluated using the agar disc diffusion method. Sterilized plate count agar (90 mL) was poured into a Petri dish and allowed to solidify. *Escherichia coli* (TISTR 527, sourced from the Thailand Institute of Scientific and Technological Research) was spread evenly across the agar surface using a sterile cotton swab under aseptic conditions. Sterile discs were placed on the agar, and 10 µL of the sample solutions (10 mg/mL) or film samples (6 mm in diameter) were applied to the discs. The plates were then incubated at 37 °C for 24 h. After incubation, the inhibition zones, including the discs, were measured in millimeters using a ruler. Each test was performed in duplicate. For the antimicrobial testing, a single *E. coli* colony was grown in 5 mL of tryptone soya broth and incubated at 37 °C for 24 h to achieve a concentration of 10^6^–10^7^ CFU/mL. Whatman filter paper discs (6 mm in diameter) were prepared and sterilized by autoclaving at 120 °C for 15 min to ensure sterility.

#### 2.3.10. Minimum Inhibitory Concentration (MIC) Assay

The antibacterial efficacy of the prepared films was evaluated using the broth microdilution method against *Escherichia coli* ATCC 25922 and *Staphylococcus aureus* ATCC 25923, each with an inoculum size of ~10^5^ CFU/mL. Penicillin G at a concentration of 16 μg/mL served as the positive control, while wells containing bacterial suspension and broth without any sample acted as the negative control, and wells with only Tryptic Soy Broth (TSB) served as the blank control. Each film was cut into very small pieces, weighed accurately, and cleaned using 30% hydrogen peroxide to remove contaminants. The hydrogen peroxide was then removed, and the films were washed thoroughly with distilled water and dried. The prepared samples were transferred to the bottom of 96-well microplates in duplicate wells. Bacterial suspension was added to each well except for the blank control wells, and positive control wells were prepared by adding Penicillin G along with the bacterial suspension. The microplate was incubated at 37 °C for 24 h, after which bacterial growth was observed visually to evaluate the antibacterial activity. Duplicate tests were performed for all samples to ensure accuracy and reproducibility.

#### 2.3.11. WVTR and OTR

PLA films were determined by measuring the water vapor transmission rate (WVTR) in accordance with the ASTM E96-93 [29] (modified) standard method. The test was carried out under a relative humidity of 75% for 24 h. Three replications were performed for each sample. The WVTR of the prepared films was calculated using Equation (2):

(2) $$WVTR\left(\frac{g-mm}{m^{2}-day}\right)=\frac{{W_{f}-W}_{i}}{t\times A}\times T$$

where $W_{i}$ and $W_{f}$ are weight of the dried sample (g) and weight of the sample after storage under saturated salt solution for 1 day (g). T is the thickness of the film (mm) while *t* and *A* are the measurement duration (day) and effective area of the film (m^2^), respectively.

The oxygen transmission rate (OTR) of PLA film was determined by using OX-TRAN^®^ model 1/50 oxygen permeability in accordance with the ASTM D3985 standard method [30]. The test was conducted under O_2_ and N_2_ atmospheres at a relative humidity of 0% and a temperature of 23 °C.

## 3. Results and Discussion

### 3.1. Morphology Study

**Figure 1:**
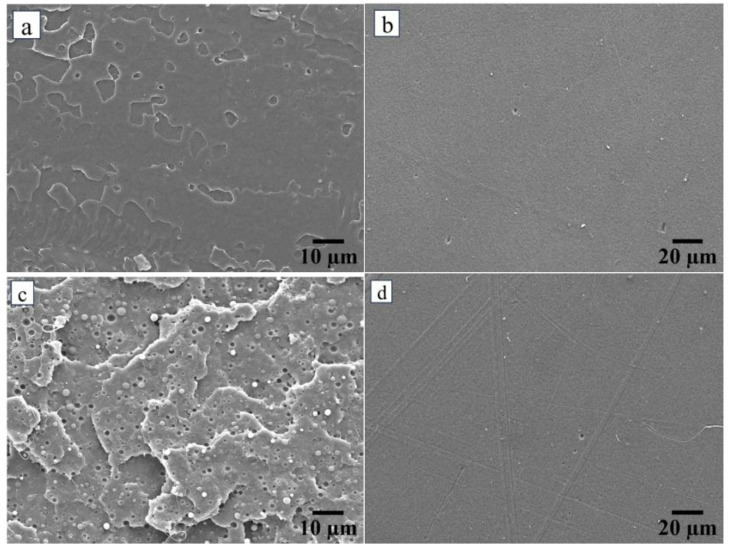

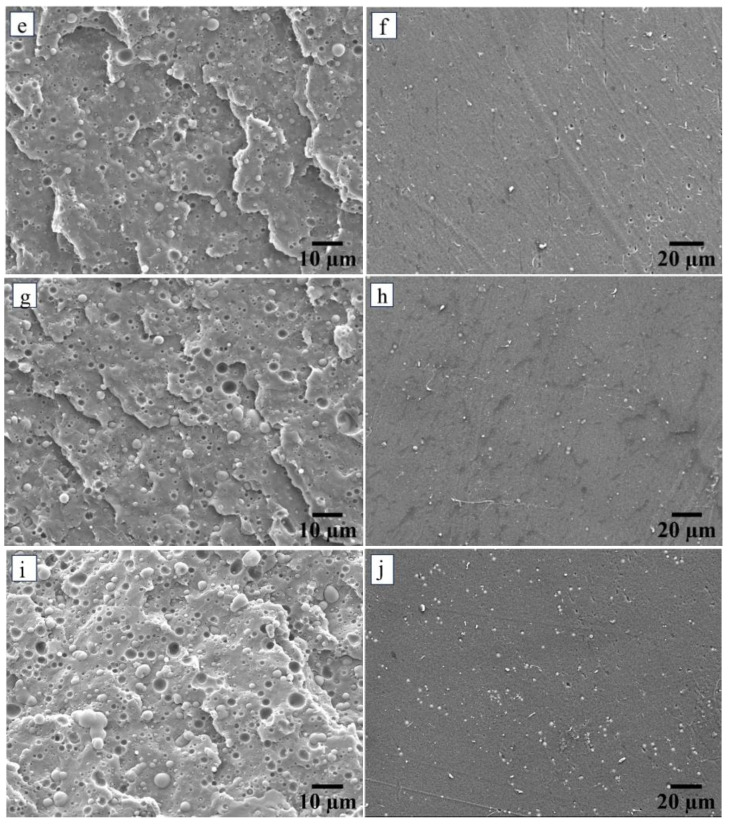
(**a**) cross-sectional SEM image of neat PLA, (**b**) SEM surface image of neat PLA, (**c**) cross-section SEM image of IMPLA, (**d**) SEM surface image of IMPLA, (**e**) cross-sectional SEM image of IMPLA2, (**f**) SEM surface image of IMPLA2, (**g**) cross-section SEM image of IMPLA5, (**h**) SEM surface image of IMPLA5, (**i**) cross-section SEM image of IMPLA7, (**j**) SEM surface image of IMPLA7.

**Figure 2:**
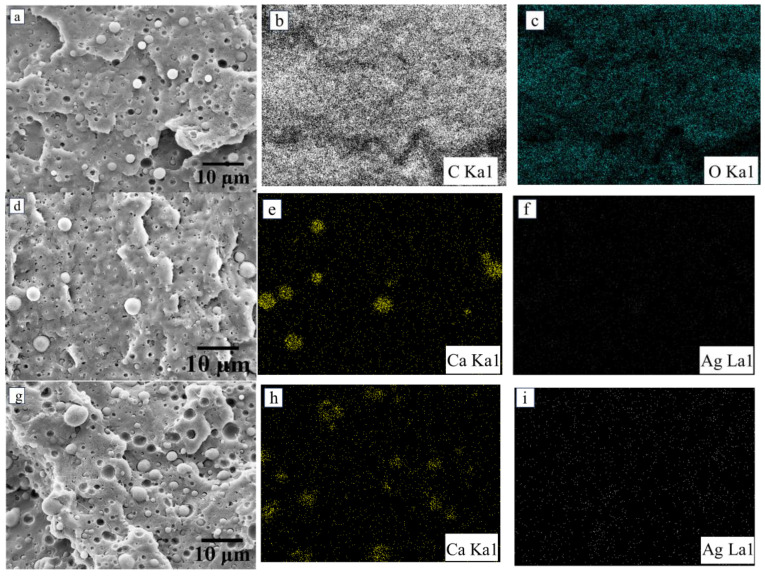

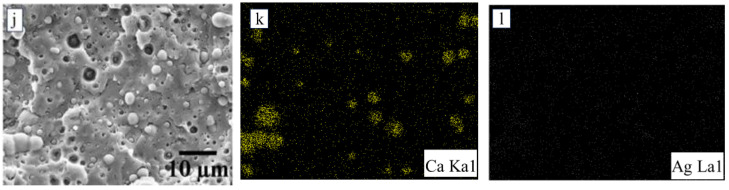
(**a**) cross-sectional SEM image of IMPLA, (**b**) carbon EDS mapping image, (**c**) oxygen EDS mapping image, (**d**) cross-sectional SEM image of IMPLA2, (**e**) calcium image mapping, (**f**) silver mapping image, (**g**) cross-sectional SEM image of IMPLA5, (**h**) calcium mapping image, (**i**) silver mapping image, (**j**) cross-sectional SEM image of IMPLA7, (**k**) calcium mapping image, and (**l**) silver mapping image.

Cryogenic cross-section and surface SEM analyses were performed to investigate the morphological characteristics of neat PLA following the inclusion of an impact modifier and the embedding of vaterite CaCO_3_-Ag hybrid microspheres. Figure 1c illustrates IMPLA, revealing impact modifier particles with a particle size distribution (PSD) ranging from 1.25 to 2.85 μm and a mean diameter (Dm) of 1.95 μm ± 0.48 μm, uniformly dispersed throughout the PLA matrix. Upon loading the vaterite CaCO_3_-Ag hybrid into IMPLA, a consistent distribution and morphology were observed. This uniformity is attributable to the similar sizes of both the impact modifier and the vaterite CaCO_3_-Ag hybrid microspheres, which are approximately equal. The PSD and D_m_ of vaterite CaCO_3_-Ag hybrid microspheres have been documented in a histogram presented in our prior publication [24]. Figure 2 displays cross-sectional and surface EDS mapping images of both IMPLA and IMPLA loaded with 2, 5, and 7 wt% vaterite CaCO_3_-Ag hybrid microspheres. While the EDS elemental analysis of the vaterite CaCO_3_-Ag hybrids aligns with our prior findings, the analysis of IMPLA loaded with vaterite CaCO_3_-Ag hybrids revealed a fluctuation in the average wt% of silver atoms in the cross-section, ranging from 0.09 to 0.25 wt% without discernible trends. These discrepancies in silver content may stem from the PLA matrix’s coverage of the hybrid microspheres, potentially influencing how the sample interacts with X-ray analysis and thus resulting in variations in the measured silver content.

### 3.2. Structural Study

**Figure 3:**
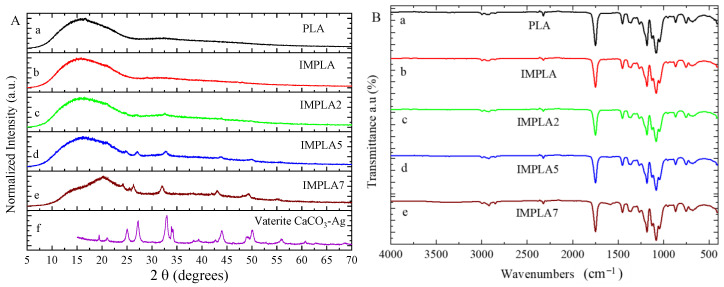
(**A**) XRD fingerprints and (**B**) ATR-FTIR spectra of (a) PLA, (b) IMPLA, (c) IMPLA2, (d) IMPLA5, and (e) IMPLA7, and (f) vaterite CaCO_3_-Ag microspheres.

Figure 3A presents the X-ray diffraction (XRD) patterns of various samples, including the neat PLA film, PLA modified with an impact modifier (IMPLA), and IMPLA containing different loadings of vaterite CaCO_3_-Ag hybrid microspheres. In Figure 3Aa, the XRD pattern of the neat PLA polymer film displays a broad peak at 2θ = 16°, characteristic of both semi-crystalline and amorphous regions within the PLA matrix [31,32]. Upon introduction of the impact modifier (IMPLA), as shown in Figure 3Ab, the XRD pattern retains the broad characteristic peak, indicating minimal structural changes in PLA due to the modifier. However, upon subsequent embedding of vaterite CaCO_3_-Ag microspheres in IMPLA, distinct sharp peaks appear at 2θ values of 25°, 27.2°, 32.8°, 34.1°, 43.9°, and 49° (Figure 3Ac). These peaks correspond to the crystalline nature of the CaCO_3_-Ag hybrid, affirming the stability of the dominant vaterite polymorph within the IMPLA polymer matrix. Furthermore, Figure 3A reveals that increasing the loading of vaterite polymorphs from 2 to 7 wt% enhances the intensity of the related peaks, indicating a proportional increase in crystalline content. The XRD fingerprint of the vaterite CaCO_3_-Ag microspheres alone is depicted in Figure 3Af, with detailed discussion available in our previous publication [24]. Briefly, it shows distinct peaks at 2θ values of 19.5°, 21°, 25°, 27.2°, 32.8°, 34.1°, 43.9°, 49°, 50.1°, and 55.8°, confirming the existence of the vaterite polymorph of CaCO_3_. Remarkably, in Figure 3Ae, the broad characteristic peak of PLA in IMPLA 7 exhibits slight sharpening, suggesting heightened crystallinity of the PLA polymer matrix with higher vaterite CaCO_3_-Ag microsphere loading. Moreover, the absence of any calcite peak in IMPLA2, IMPLA5, and IMPLA7 indicates the stability of the vaterite polymorph during the micro-compounding process, with no observed transformation in crystalline polymorphs.

Figure 3B depicts the infrared spectra of the neat PLA film, IMPLA, and IMPLA with 2, 5, and 7 wt% CaCO_3_-Ag microspheres loading. As shown in Figure 3Ba, the spectrum of neat PLA exhibits characteristic stretching for C=O at 1746 cm^−1^ and CH_3_ (asymmetric and symmetric) at 2995 and 2946 cm^−1^. Additionally, bending frequencies for CH_3_ (asymmetric and symmetric) are identified at 1452 and 1361 cm^−1^, respectively. The bands at 1079 and 1182 cm^−1^ are attributed to C–O in O-C=O and C-O in CH–O stretching vibrations, respectively [33]. The spectrum of vaterite CaCO_3_-Ag microspheres has been reported in our previous publication [23]. Remarkably, upon introducing the impact modifier and loading vaterite CaCO_3_-Ag microspheres into the PLA polymer matrix, no discernible changes are observed in the spectrum. This observation suggests remarkable compatibility between the impact modifiers and vaterite CaCO_3_-Ag microspheres, as well as their uniform distribution within the PLA matrix without notable alterations to its molecular structure. Additionally, PLA may effectively coat the vaterite CaCO_3_-Ag microspheres, resulting in the dominance of PLA spectra in the composite material. Therefore, despite incorporating vaterite CaCO_3_-Ag microspheres, the PLA spectra remain largely unchanged. This absence of spectral changes suggests that there is no significant chemical interaction between the microspheres and PLA, further highlighting the compatibility and stability of the system.

It was reported that the interaction between carboxyl groups of poly(methacrylic acid) and bivalent metallic ions of Zn^2+^ was observed via high-resolution solid-state ^13^C NMR spectroscopy [34]. Kumar et al. [35] suggested that bivalent ions of Ca^2+^ are possibly coordinated with the carboxy groups of PLA, in which the contents of nano calcium carbonate of PLA composites are 3–7 wt%. In addition, from their study, the characteristics of calcium carbonate FTIR peaks were obscured by the vibration band of PLA; therefore, the vibrations indicating the carboxyl group and Ca^2+^ interaction were not observed. Moreover, the degree of the vibrations derived from the interaction would be lower than the detection limit of the FTIR instrument.

### 3.3. Synchrotron X-Ray Absorption Spectroscopy (XAS)

**Figure 4:**
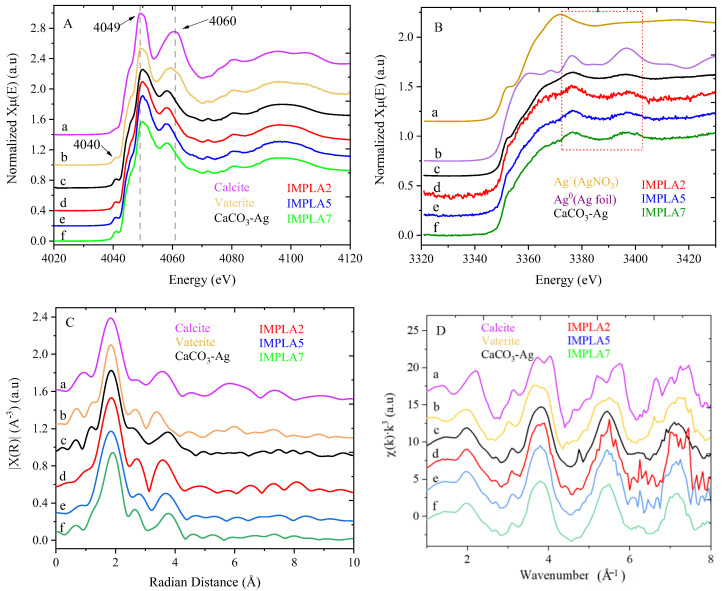
(**A**) Ca K-edge X-ray absorption near edge structure (XANES), (**B**) Ag-L_3_ edge XANES spectra, (**C**) R-space Ca K-edge extended X-ray absorption fine structure (EXAFS) spectra, and (**D**) k-space Ca K-edge extended X-ray absorption fine structure (EXAFS). Spectra are labeled as follows: (a) Calcite, (b) Vaterite, (c) CaCO₃–Ag, (d) IMPLA2, (e) IMPLA5, and (f) IMPLA7.

Ca K-edge X-ray absorption near edge structure (XANES) spectra of vaterite CaCO_3_-Ag hybrid microspheres and IMPLA containing 2, 5, and 7 wt% vaterite CaCO_3_-Ag are shown in Figure 4A, alongside vaterite and calcite references. The XANES reference spectra of calcite and vaterite reveal distinct differences. Calcite exhibits only three peaks: a white line peak at 4049 eV (1s → 4p transition), a shoulder peak at 4045 eV (assigned to the 1s→4s transition), and a post-edge feature at 4061 eV. In contrast, vaterite displays additional features, including a prominent pre-edge peak at 4040 eV (corresponding to the 1s→3d transition of first-row transition metals), a shoulder peak at 4045 eV, a white line peak at 4049 eV, and a post-edge peak at 4059 eV. This additional pre-edge peak in vaterite’s spectrum indicates the polymorphic transformation from calcite to vaterite and highlights the unique electronic structures and symmetries of these two CaCO_3_ polymorphs [36]. This same pre-edge peak at 4040 eV appears in the synthesized vaterite CaCO_3_-Ag microspheres (Figure 4Ac) and in the three samples (IMPLA2, IMPLA5, and IMPLA7), confirming the existence of CaCO_3_ in the vaterite polymorph. These results indicate the stability of the vaterite CaCO_3_-Ag microspheres during processing after embedding in the PLA polymer matrix. Furthermore, notable differences exist in the post-edge peak of CaCO_3_-Ag compared to CaCO_3_ in the vaterite polymorph, with the intensity of this peak being lower and slightly shifted to 4058 eV, suggesting variations in the local chemical environment around the Ca ions due to the presence of AgNPs.

Ag L_3_-edge XANES analysis probes the unoccupied d or s orbital states by creating a core hole at the 2p_3/2_ level, providing detailed information on the electronic structure and oxidation states of AgNPs [37]. Figure 4B illustrates the spectra of AgNPs in vaterite CaCO_3_-Ag hybrid and after embedding in PLA samples, alongside Ag foil (representing Ag^0^) and AgNO_3_ (representing Ag^+^) as references. The XANES spectra of the vaterite CaCO_3_-Ag microspheres in Figure 4B exhibit characteristic peaks of both Ag^0^ and Ag^+^, indicating the coexistence of both species of AgNPs, as supported by the reference spectra. This aligns with our previous XPS analysis, which confirmed the existence of both Ag^0^ and Ag^+^, with Ag^0^ being the predominant species [23]. Upon loading the vaterite CaCO_3_-Ag microspheres in PLA, the XANES features remain unchanged, indicating the continued coexistence of both Ag^0^ and Ag^+^ species. This is an interesting result because, based on our previous XPS analysis [23,24], we found that after embedding the vaterite CaCO_3_-Ag microspheres in polyvinyl alcohol (PVA) and carboxymethyl cellulose (CMC) via solution casting, Ag^0^ converted to Ag^+^, with no remaining Ag^0^. This may indicate that the absence of water in non-solution mixing, such as melt blending, prevents the conversion of Ag^0^ to Ag^+^ ions, possibly due to the lack of a solvent environment that promotes oxidation.

To gain insights into the neighborhood environment of calcium ions, including coordination number and distances to neighboring atoms, extended X-ray absorption fine structure (EXAFS) analysis was conducted. The Ca K-edge EXAFS spectra of vaterite CaCO_3_-Ag hybrid microspheres and IMPLA containing 2, 5, and 7 wt% vaterite CaCO_3_-Ag hybrid, along with vaterite and calcite references, were plotted in both R space (Figure 4C) and k space (Figure 4D). The major peak in the Fourier transform R space observed in all samples corresponds to a first shell of Ca-O at around 1.84 Å (without phase shift correction). The broad peak at 3.4 Å corresponds to Ca-Ca scattering.

The EXAFS spectra of vaterite and calcite references were fitted according to the lattice parameters and space group of vaterite (hexagonal P3_2_21) [38] and calcite (trigonal R3c space group) [39] polymorphs using ARTEMIS. Table 2 presents the EXAFS fitting results for all samples. The first coordination shell of Ca-O distances, with six nearest neighbor oxygen atoms around calcium, shows that calcite and vaterite have similar distances of approximately 2.35 Å. In contrast, CaCO_3_-Ag, IMPLA2, IMPLA5, and IMPLA7 exhibit shorter Ca-O distances. The Ca–C bond distances in CaCO_3_-Ag, IMPLA2, IMPLA5, and IMPLA7 are also shorter than those in calcite and vaterite. The changes in bond length distances corroborate the differences observed in the XANES spectral features at the post-edge region, indicating that AgNPs modify the electronic structure of CaCO_3_ by donating or accepting electrons from other molecules or materials through redox reactions. The introduction of AgNPs to CaCO_3_ affects its morphology, potentially leading to the formation of hybrid particles with AgNPs embedded within the CaCO_3_ matrix. The resulting hybrid material, comprising AgNPs trapped inside CaCO_3_ microparticles, could induce a transformation of CaCO_3_ into vaterite, resulting in denser packing.

### 3.4. Cell Viability

**Figure 5:**
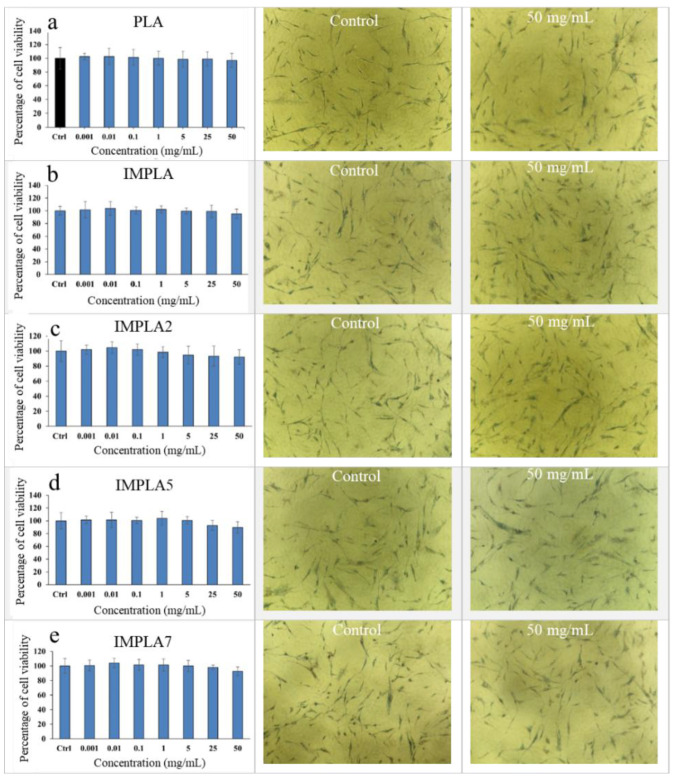
Human dermal fibroblast (HDF) cytotoxicity test by MTT assay and microimages of water-insoluble purple formazan crystals in cells (control and in the presence of 50 mg/mL of each sample). (**a**) PLA, (**b**) IMPLA, (**c**) IMPLA2, (**d**) IMPLA5 and (**e**) IMPLA7.

The assessment of cytotoxicity is integral to the evaluation of products used in both the food industry and biomedical applications. Our recent publication [24] discusses the toxicity profiles of vaterite CaCO_3_-Ag hybrid microspheres at varying concentrations in detail. Additionally, we conducted an MTT assay on neat PLA, IMPLA, and their composite films containing different proportions of vaterite CaCO_3_-Ag microspheres, utilizing a human dermal fibroblast (HDF) cell culture. The results, as illustrated in Figure 5, reveal that the prepared films exhibit significant cytocompatibility with human cells and demonstrate complete biocompatibility, even at concentrations up to ≤50 mg/mL, without any observable signs of toxicity. Figure 5 also includes microscopic images of HDF cells under two conditions: without samples (control) and in the presence of samples at a concentration of 50 mg/mL. These images distinctly depict the thriving HDF cells, even in the presence of high sample concentrations, conclusively affirming the full biocompatibility of the tested samples, with no indications of toxicity.

### 3.5. Antimicrobial Test

**Figure 6:**
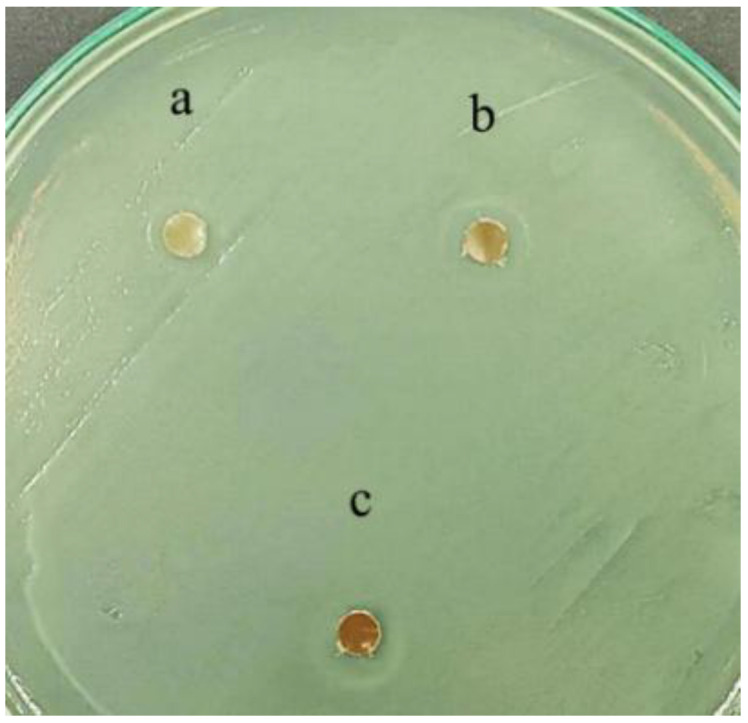
Antimicrobial inhibition zone of (a) IMPLA2, (b) IMPLA5, and (c) IMPLA7.

The antimicrobial activity of the samples was evaluated using the disc diffusion method against *Escherichia coli* (*E. coli*). Despite the known strong antimicrobial properties of CaCO_3_-Ag microspheres, no distinct inhibition zones were observed for any of the samples. Instead, a haze-like zone was noted around the discs for all three samples, as shown in Figure 6. The lack of clear inhibition zones can be attributed to the limited diffusion ability from the PLA matrix through the agar hydrogel structure. As a result, the CaCO_3_-Ag microspheres remain localized within the PLA matrix and do not effectively migrate into the agar medium. This restricted diffusion likely causes the observed haze-like appearance, indicating some degree of antimicrobial activity but without the formation of well-defined inhibition zones. It is interesting to note that clear inhibition zones are more likely to be observed when using a water-soluble polymer matrix. Vaterite CaCO_3_-Ag microspheres possess strong antimicrobial properties against both *E. coli* and bacteria extracted from pork, as reported in our previous publication [23]. However, after embedding in PLA, their diffusion into the agar is restricted, which limits their effectiveness in the disc diffusion test. Additionally, a study by Kourmouli et al. [40] indicated that the antibacterial behavior of AgNPs is largely due to the ions they release upon oxidation and dilution in aqueous solutions. Therefore, the Kirby-Bauer method and similar tests can be used to assess their antimicrobial activity based on their ability to release ions.

To further assess the antimicrobial properties, we performed the minimum inhibitory concentration (MIC) test, which offers a distinct advantage over the disc diffusion method as it directly evaluates the antibacterial effect without being influenced by the diffusion properties of the material or the medium [41]. The MIC results demonstrated that the samples IMPLA2, IMPLA5, and IMPLA7 effectively inhibited the growth of both gram-positive and gram-negative bacteria, with IMPLA7 being the most effective. IMPLA7 exhibited an MIC value of less than 0.067 g/mL, significantly lower than that of IMPLA2 and IMPLA5, indicating its superior antibacterial efficacy. Similarly, samples IMPLA2 and IMPLA7 showed inhibitory effects against both types of bacteria, although their efficacy was lower compared to IMPLA7. These findings, summarized in Table 3, confirm the strong antimicrobial potential of the tested films, particularly IMPLA7, under conditions where direct contact between the film and bacteria is ensured.

### 3.6. Macromechanical and Nanomechanical Properties

**Figure 7:**
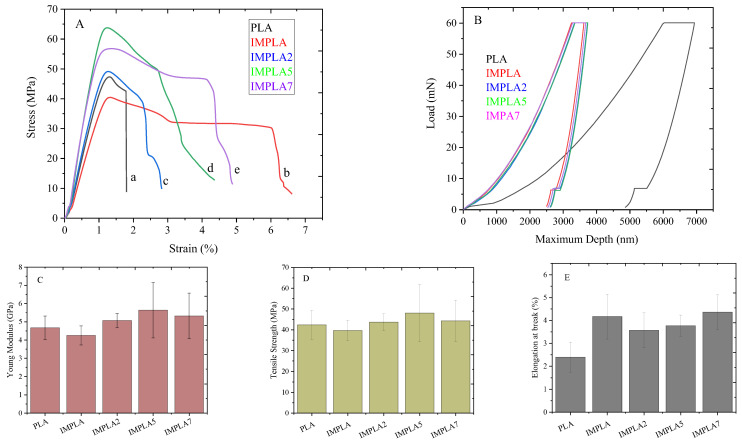
(**A**) Tensile stress-strain curves of (a) neat PLA, (b) IMPLA, (c) IMPLA2, (d) IMPLA5, (e) IMPLA7, (**B**) Load−displacement curves for PLA, IMPLA, IMPLA2, IMPLA5, and IMPLA7 from nanoindentation test, (**C**) Young’s modulus bar graph, (**D**) Tensile strength bar graph and (**E**) Elongation at break bar graph.

Figure 7A shows the stress-strain curves of neat PLA, IMPLA, IMPLA2, IMPLA5, and IMPLA7. Figure 7Ab shows that IMPLA has a lower stiffness and strength than neat PLA. However, as shown in Figure 7Ac, both the stiffness and strength improved in IMPLA2 and further reached to highest in the IMPLA5 sample. Figure 7C–E show the bar graphs of Young’s modulus, tensile strength, and elongation at break, respectively. Furthermore, the effect of impact modifier and vaterite CaCO_3_-Ag microspheres loading on the strength, modulus, elongation at the break, and toughness of PLA composite films is presented in Table 4. From Table 4, it can be observed that the modulus and tensile strength of neat PLA decreased from 4.67 ± 0.64 GPa and 42.34 ± 6.99 MPa to 4.25 ± 0.53 GPa and 39.68 ± 4.89 MPa, respectively, with 5 wt% impact modifier loading. However, the elongation at break improved from 2.39 ± 0.65% to 4.17 ± 0.98%, indicating enhanced ductility of IMPLA. Furthermore, with the embedding of 2 wt% vaterite CaCO_3_-Ag microspheres, the modulus and strength increased to 5.07 ± 0.38 GPa and 43.65 ± 4.07 MPa, respectively. However, the elongation at break slightly decreased and reached 3.57 ± 0.76%. The modulus and strength continued to increase with embedding 5 wt% vaterite CaCO_3_-Ag microspheres and reached a maximum of 5.63 ± 1.51 GPa and 48.07 ± 13.81 MPa, respectively. However, with the embedding of 7 wt% of vaterite CaCO_3_-Ag microspheres, the strength and modulus decreased slightly to 5.32 ± 1.24 GPa and 44.27 ± 9.93 MPa, respectively. This downturn may be attributed to a weaker vaterite CaCO_3_-Ag/PLA matrix interface due to the higher surface area of the microspheres in higher loading, consequently decreasing the strength and modulus. It is worth mentioning that despite this decrease, the strength and modulus of the composite film are still higher than those of the IMPLA film. Moreover, the elongation at break slightly decreased with the embedding of vaterite CaCO_3_-Ag microspheres and increased with increased vaterite CaCO_3_-Ag microspheres loading. The toughness of the filled IMPLA film increased with increasing microsphere content and was higher than that of unplasticized PLA. The modulus and strength of PLA films filled with vaterite CaCO_3_-Ag microspheres, at 5 GPa and higher than 40 MPa, respectively, are comparable to other food packaging films derived from PLA [42,43] and PE packaging film [44]. However, elongation at break of the filled PLA is limited to 3.5–4.5%; the film is not suitable for highly stretchable films.

To explore the nanomechanical properties, nanoindentation tests were performed on the neat PLA film as well as on compositions containing impact modifier and vaterite CaCO_3_-Ag hybrid microspheres. The load−displacement curves, depicted in Figure 7B, allowed determination of the reduced modulus (E_r_) and hardness (H) of the prepared films. The values for E_r_ and H are provided in Table 4. Both the impact modifier and the incorporation of vaterite CaCO_3_-Ag microspheres into PLA resulted in shifts of the curves towards lower penetration depths, indicating enhanced resistance to penetration and improved deformability resistance of the PLA film against the indenter. These improved deformability and enhanced resistance to penetration are consistent with the macro-mechanical properties observed via universal testing machine analysis. This alignment underscores the effectiveness of the incorporated impact modifier and vaterite CaCO_3_-Ag hybrid microspheres in enhancing the mechanical properties of the PLA film, thereby validating their potential for practical applications.

### 3.7. Thermal Properties

**Figure 8:**
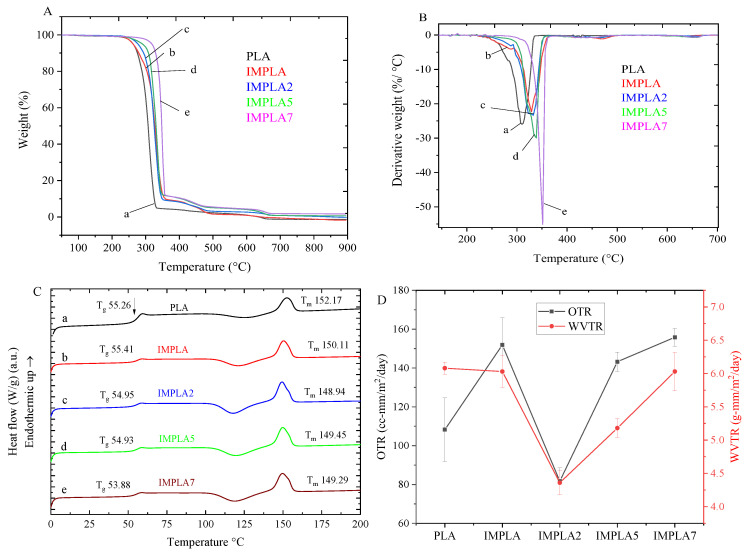
(**A**) TGA, (**B**) DTG, and (**C**) DSC plots, and (**D**) OTR and WVTR plots of (a) neat PLA, (b) IMPLA, (c) IMPLA2, (d) IMPLA5, and (e) IMPLA7.

The thermal behavior of neat PLA and its composite films, created by blending with an impact modifier and vaterite CaCO_3_-Ag hybrids, was analyzed using TG-DTG methods. These films are intended for food packaging, so ensuring their ability to withstand processing and storage temperatures is crucial. Thermal stability data from TGA analysis ensures that the packaging materials remain stable and do not release active substances when exposed to high temperatures, thus maintaining the safety and quality of the packaged food. Figure 8A,B show the TG-DTG plots. Neat PLA film exhibited a single major weight loss stage, beginning at 288.4 °C, attributed to the decomposition of PLA backbone chains [45]. The composite film with impact modifier (IMPLA) showed two stages of thermal degradation with onset temperatures at 299 °C and 383 °C, corresponding to weight losses of 90.4% and 7.7%, respectively. It is attributed to the decomposition of PLA and impact modifier. The addition of vaterite CaCO_3_-Ag microspheres at 2%, 5%, and 7% wt in PVA film resulted in three stages of degradation. For IMPLA2, the major stage (90.9%), 2nd stage (5.7%), and 3rd stage (2.4%) of weight loss occurred at onset temperatures of 305 °C, 379 °C, and 525 °C, respectively. The additional stage compared to IMPLA is attributed to the decomposition of vaterite CaCO_3_ to CaO [46]. For IMPLA5 and IMPLA7, the major stage (88.1% and 88.2%), 2nd stage (6.1% and 6.0%), and final stage (4.0% and 3.4%) of degradation occurred at onset temperatures of 317 °C and 346 °C, 386 °C and 380 °C, and 536.3 °C and 528 °C, respectively. The maximum degradation temperature (T_max_) of neat PLA polymer film was 308.7 °C, which increased gradually upon mixing with the impact modifier and embedding of vaterite CaCO_3_-Ag, reaching 350 °C in IMPLA7 (Table 5).

The DSC thermograms of the films are shown in Figure 8C. DSC analysis is essential for evaluating PLA food packaging films as it precisely determines the glass transition temperature (T_g_), which is critical for designing materials that maintain stability and functionality during storage and transportation. The DSC curve for neat PLA polymer exhibited a T_g_ at 55.26 °C and a melting temperature (T_m_) at 152.17 °C, reflecting its semicrystalline nature. However, the T_g_ of the films gradually shifted to lower temperatures with increasing loading of vaterite CaCO_3_-Ag microspheres, reaching a minimum of 53.88 °C in IMPLA7. This shift indicates no chemical reaction occurred between vaterite CaCO_3_-Ag microspheres and PLA, suggesting an increase in the free volume of the PLA macromolecular chains, making them more mobile. These results align well with ATR-FTIR spectroscopy findings. Furthermore, the T_m_ slightly decreased, which may be due to changes in the amorphous-crystalline phases within the PLA polymer matrix. The slight decrease in T_m_ is likely due to the formation of less perfect or smaller crystalline regions, which require less thermal energy to melt [47]. The crystallization (T_c_) is observed at 125 °C. The degree of crystallinity (X_c_) of PLA and its composites with vaterite CaCO_3_-Ag was calculated and summarized in Table 5. The X_c_ of the PLA polymer matrix increased from 7.1% in neat PLA to 28.8% in IMPLA7, giving rise to a reduced brittle amorphous phase at room or freezing temperature. These results are consistent with XRD findings. The increase in crystallinity can be attributed to the nucleating effect of the CaCO₃-Ag microspheres, which promote the orderly arrangement of PLA chains during cooling [48]. Higher crystallinity in PLA food packaging can contribute to enhanced mechanical properties and thermal stability, improving the overall performance and durability of the packaging material.

### 3.8. OTR and WVTR

The use of PLA as a barrier against gases, vapors, and organic compounds is limited compared to certain petroleum-based polymers. This limitation could potentially restrict its application in packaging scenarios that require high barrier properties [49]. Incorporating inorganic compounds into PLA can notably enhance its barrier properties. Figure 8D illustrates both the oxygen transmission rate (OTR) and water vapor transmission rate (WVTR) plots of the samples, while Table 5 presents the OTR and WVTR values of the PLA films prepared in this study. The neat PLA films exhibited an OTR exceeding 100 cc-mm/m^2^/day, which significantly increased to over 150 cc-mm/m^2^/day after mixing with impact modifier (IMPLA). However, upon loading 2 wt% CaCO_3_-Ag microspheres, the OTR notably decreased to approximately 80 cc-mm/m^2^/day. This OTR falls within the range reported in literature for packaging materials such as PP or PE, where 50–100 cc-mm/m^2^/day is deemed suitable for less oxygen-sensitive products, such as snacks and dry foods [50]. Additionally, the lowest WVTR was achieved in the IMPLA2 film (4.36 ± 0.18 g-mm/m^2^/day), which aligns with its OTR result, which was the lowest as presented in Table 5. The industry standard for WVTR in food packaging materials, including PLA, typically falls within the range of 1 to 10 g-mm/m^2^/day [51].

## 4. Conclusions

Biocompatible and antimicrobial PLA composite films were successfully prepared via a melt mixing method by introducing biogenic vaterite CaCO_3_-Ag hybrid microspheres. Structural analysis confirmed the stability and compatibility of the hybrid microspheres within the PLA matrix, with minimal impact on the polymer’s molecular structure. The composite films exhibited significant biocompatibility, improved mechanical properties, and enhanced thermal stability. While the antimicrobial efficacy observed through the disc diffusion method was limited by the restricted diffusion of the CaCO_3_-Ag microspheres within the PLA matrix, the MIC results demonstrated strong antimicrobial efficacy, likely due to the direct contact between the film and the bacteria. The improved barrier properties against oxygen and water vapor further emphasize the suitability of these films for food packaging applications. Overall, the PLA-vaterite CaCO_3_-Ag hybrid films present a promising approach to developing sustainable and effective food packaging materials, contributing to environmental sustainability and food safety.

## Figures and Tables

**Table 1:** Sample codes and corresponding wt% of PLA, impact modifier, and CaCO₃-Ag hybrid microspheres in prepared films.

Samples	Elvaloy^®^ PTW (wt%)	CaCO_3_-Ag Hybrid (wt%)	PLA (wt%)
IMPLA	5	0	95
IMPLA2	5	2	93
IMPLA5	5	5	90
IMPLA7	5	7	88

**Table 2:** Ca K-edge EXAFS fitting results.

Sample	Scatting Path (Å)					SO_2_	D*E*_0_	R-Factor
	Ca-O	Ca-C	Ca-O-C	Ca-O	Ca-Ca			
	N = 6	N = 6	N = 12	N = 6	N = 6			
Calcite	2.350	3.340	2.915	3.541	3.981	0.747	3.526	0.0129
Vaterite	2.357	3.167	3.158	3.569	4.053	0.776	2.422	0.0199
CaCO_3_-Ag	2.316	2.971	3.474	3.626	4.056	0.747	−1.669	0.0115
IMPLA2	2.311	2.973	3.366	3.603	4.050	0.885	−1.686	0.0077
IMPLA5	2.314	2.951	3.291	3.612	4.047	0.898	−1.877	0.0060
IMPLA7	2.325	2.983	3.360	3.637	4.089	0.747	−0.253	0.0081

Remark: σ^2^ for all fits is less than 0.03. N = the coordination number. SO_2_ = Amplitude reduction, Δ*E*_0_ = the shift in the Ca K-edge energy. The k-space data range was from 3.6 to 4.1 Å^−1^.

**Table 3:** Minimal inhibitory concentration (g/mL) of films against gram-positive and gram-negative bacteria.

Sample	MIC (g/mL)	
	*Staphylococcus aureus*	*Escherichia coli*
IMPLA2	0.267	0.133
IMPLA5	0.133	≤0.067
IMPLA7	≤0.067	≤0.067

**Table 4:** Macromechanical and nanomechanical properties.

Sample	CaCO_3_-Ag Content (wt%)	Young’s Modulus (GPa)	Tensile Strength (MPa)	Elongation at Break (%)	Toughness (J/m^3^)	Reduced Modulus (GPa)	Hardness (GPa)
PLA	0	4.67 ± 0.64	42.34 ± 6.99	2.39 ± 0.65	61.90 ± 9.89	4.870 ± 0.111	0.200 ± 0.009
IMPLA	0	4.25 ± 0.53	39.68 ± 4.89	4.17 ± 1.62	109.11 ± 50.67	5.198 ± 0.171	0.228 ± 0.010
IMPLA2	2	5.07 ± 0.38	43.65 ± 4.07	3.57 ± 0.76	98.39 ± 23.04	5.096 ± 0.055	0.216 ± 0.002
IMPLA5	5	5.63 ± 1.51	48.07 ± 13.81	3.77 ± 0.46	128.82 ± 36.26	5.097 ± 0.038	0.212 ± 0.005
IMPLA7	7	5.32 ± 1.24	44.27 ± 9.93	4.36 ± 0.76	141.98 ± 40.44	5.189 ± 0.131	0.214 ± 0.008

**Table 5:** Thermal properties and permeability parameters.

Sample	T_g_ (°C)	T_m_ (°C)	ΔH (J/g)	Crystallinity (X_c_,%)	T_max_ (°C)	WVTR (g-mm/m^2^/day)	OTR (cc-mm/m^2^/day)
PLA	55.3	152.2	6.63	7.1	308.7	6.08 (±0.09)	108.23 (±16.34)
IMPLA	55.4	150.1	19.69	22.1	330.8	6.03 (±0.24)	151.87 (±13.95)
IMPLA2	54.9	148.9	23.92	27.5	325.1	4.36 (±0.18)	81.68 (±6.82)
IMPLA5	54.9	149.5	21.76	25.8	337.8	5.18 (±0.14)	143.21 (±4.98)
IMPLA7	53.9	149.3	23.67	28.8	350.0	6.03 (±0.29)	155.78 (±4.64)

## Data Availability

The data presented in this study are available on request from the corresponding author. The data are not publicly available due to legal and privacy concerns. However, they may be made available from the corresponding author upon reasonable request and subject to appropriate data sharing agreements.
